# The effect of soil environmental factors on the yield and quality of *Pueraria lobata*

**DOI:** 10.1038/s41598-023-45918-2

**Published:** 2023-10-31

**Authors:** Fahuo Li, Yuting Li, Jianming Huang, Jingying Li, Dong Xiao, Yong Li, Longfei He, Ai-qin Wang

**Affiliations:** 1 National Demonstration Center for Experimental Plant Science Education, College of Agriculture, Guangxi University, Nanning, 530004 People’s Republic of China; 2 College of Electronics and Information Engineering, Beibu Gulf University, Qinzhou, 53501 People’s Republic of China; 3 Qinbei Rural Grassroots Organization and Construction Service Center, Qinzhou, 535000 People’s Republic of China; 4 Institute of Vegetable Research, Guangxi Academy of Agricultural Sciences, Nanning, 530007 People’s Republic of China; 5 Guangxi Key Laboratory for Agro-Envirorment and Agro-Product Safety, Nanning, 530004 People’s Republic of China

**Keywords:** Ecology, Microbiology, Plant sciences

## Abstract

*Pueraria lobata* is a typical medicinal and edible plant with great market value and demand, thus exploring the relationship between soil environmental factors and the yield and quality of *Pueraria lobata* is of great significance for its high-value cultivation. In this study, using the Guige 1 variety (*Pueraria montana var. Thomsonii*) selected by our research group as the material to compare the effects of five soil types, endophytes in three parts of *Pueraria lobata* and two fertilizers on its yield and quality. The results showed that the comprehensive evaluation effect of five soil types on the yield and quality of Guige 1 was as follow: red-yellow mixed soil (RYMS) > black loam soil (BLS) > sandy loam soil (SLS) > sandy loam soil waterlogging (SLSW) > yellow soil compaction soil (YSCS); the descending order of endophyte types and quantities is in BLS > RYMS > SLS > YSC > SLSW; applying General Compound Fertilizers (GCF) in RYMS is more suitable for the rapid expansion of Guige 1 than Organic-Slow-Release-Fertilizers (OSRF). The high potassium content in RYMS and high effective phosphorus content in BLS are positively correlated with the content of starch and isoflavone in *Pueraria lobata*. The conclusion is that the high potassium and available phosphorus content in RYMS and BLS, as well as the rich types and quantities of endophytic bacteria, are positively correlated with the yield and quality of *Pueraria lobata*. The research results have important guiding significance for the high-value cultivation of *Pueraria lobata*.

## Introduction

*Pueraria lobata* is a plant of the Pueraria genus in the subfamily Papilioideae of the Leguminosae family, which is enriched with many effective substances, such as polysaccharides, starch, saponins, puerarin, isoflavones, amino acids, daidzein, triterpenoid alkaloids and so on^1^. *Pueraria lobata* is a typical medicinal and food homologous plant, with the function of anti-inflammatory, antioxidant, hypoglycemic, alcohol and liver protection, promoting fluid production, quenching thirst, relieving muscle and fever, promoting meridians, stopping diarrhea and so on^2–4^. *Pueraria lobata* is mainly used in traditional Chinese medicine for the treatment of "eliminate thirst", hypoglycemic, anti-inflammatory, alcohol poisoning, stroke and hemiplegia, strong pain in the neck and back, measles opacity, external fever and headache and so on, while in western medicine, *P. lobata* is often used to prevent cardiovascular and cerebrovascular sclerosis, improving immunity, lowering blood pressure and blood lipids, alleviating angina and fighting cancer^5–10^. Due to the healthcare functions of isoflavones and puerarin compounds, the contents of them have also become important indicators for evaluating the quality of *P. lobata*. In Japan, the contents of daidzein, puerarin, and daidzein are indicators to evaluate the quality of *P. lobata*^11^. In the Chinese Pharmacopoeia 2000 edition: it is required that the amount of puerarin in *Pueraria montana var. lobata* should not be less than 2.4%, while that in *Pueraria montana var. thomsonii* should not be less than 0.30%. Research has shown that there are significant differences in the content of puerarin among different varieties and origins of *P. lobata*, with the content of puerarin in *Pueraria montana var. lobata* being 8-10 times higher than that in *Pueraria montana var. Thomsonii*^12^. There were significant differences in total flavonoid content and puerarin content among different varieties of *P. lobata*, among which *Pueraria montana var. lobata* being higher than that in *Pueraria montana var. Thomsonii*^13^*.*

Soil is the foundation on which all plants rely for survival. Different types of soil, due to differences in the internal texture (such as mechanical resistance, porosity, et al.) and environment, affect the exchange of water, air and nutrients between soil and plants, and ultimately impact on the growth, development, yield and quality of plants^14,15^.The nutrients in the soil affect the photosynthesis, growth and development of plants^16^, as well as the formation of secondary metabolites, which in turn influence the quality of medicinal materials^17^. During the long evolutionary process, some soil microorganisms have entered the host plant and formed a special mutually beneficial symbiotic relationship with the host^18^. Through nitrogen fixation, disease prevention, phosphorus dissolution, iron carriers, inhibition of pathogenic fungi, induction of systemic resistance and production of plant hormones, they promote the growth, enhance the stress resistance and promote the accumulation of secondary metabolites of the host plant, and thereby affect the yield and quality of the host plant, which lead this type of microorganism to be called plant growth promoting bacteria^19–21^.

Currently, *P. lobata* is a kind of medicinal and edible homologous plant producing the most isoflavones and puerarin, with enormous development and utilization value. In China, the main production area of *P. lobata* is Guangxi, with red soil, yellow soil, and lateritic red soil as the main soil types. The soil in Guangxi has the characteristics of high degree of silicon and aluminum removal, poor fertilizer retention performance, strong soil acidity, and moderate to lower fertility, which restrict the development of *P. lobata* industry. This research used the main cultivated variety of Pueraria montana var. thomsonii in Guangxi, Guige 1, as the material to analyze the effects of five soil types (red-yellow mixed soil, RYMS; black loam soil, BLS; sandy loam soil, SLS; sandy loam soil waterlogging, SLSW; yellow soil compaction soil, YSCS), two fertilizer types (General Compound Fertilizer, GCF; Organic Slow Release Fertilizers, OSRF), and endophyte populations on the yield and quality of *P. lobata*, as well as the correlation analysis among these factors. This provides a theoretical basis for the development and utilization of *P. lobata* resources and endophyte resources.

## Results

### The effect of five soil types on the yield and variety of Guige 1

**Figure 1:**
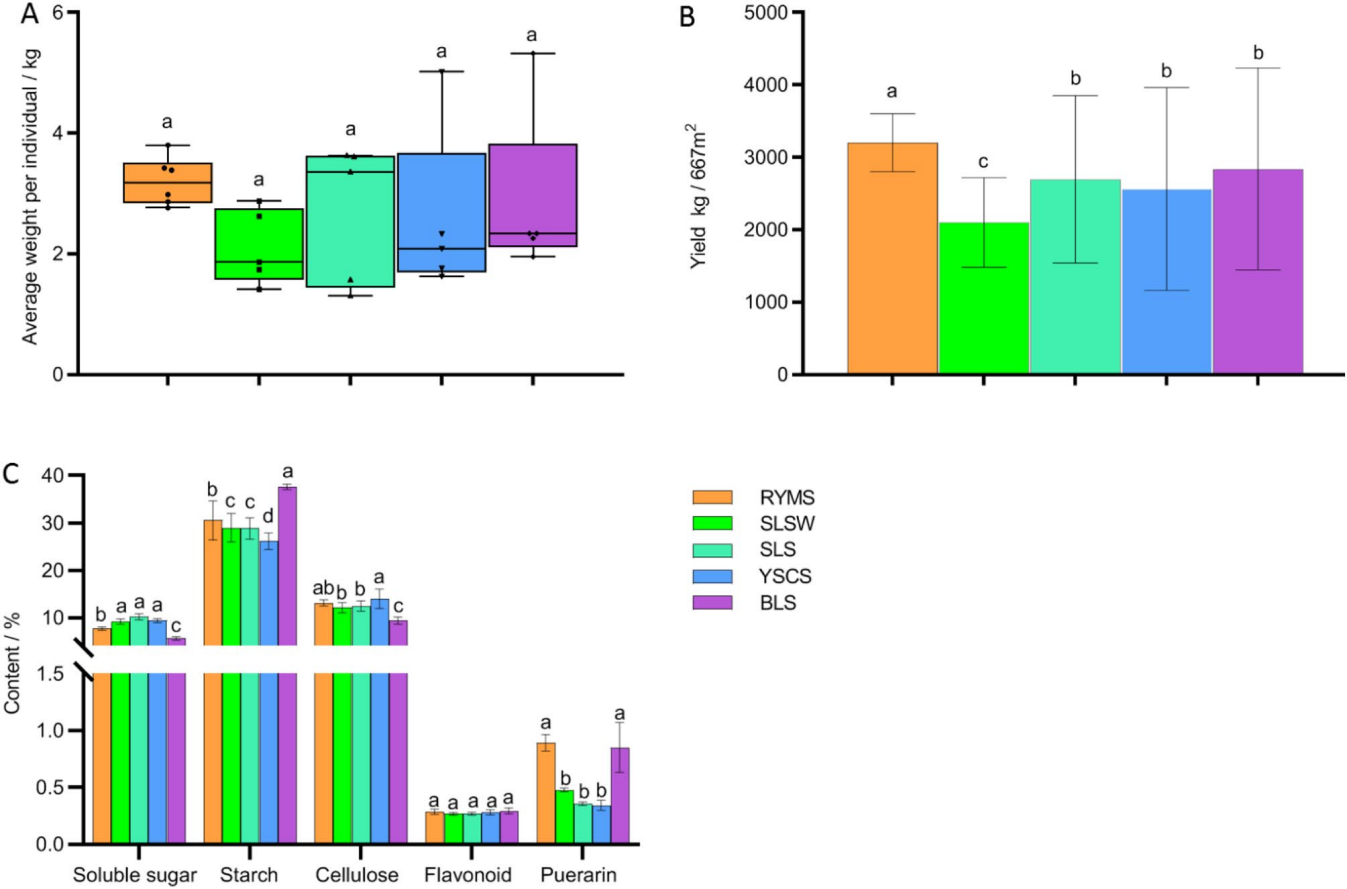
The impact of five soil types on the yield and quality of Guige 1. (**A**) The impact of five soil types on the average plant weight of Guige 1. (**B**) The impact of five soil types on the yield of Guige 1; (**C**) The contents of soluble sugar, starch, cellulose, total flavonoids and puerarin in Guige 1 in five different soil types. Different lowercase letters and “*” indicate significant difference (*p* < 0.05).

Figure 1 shows the effects of five soil types on the yield and quality of Guige 1. In Fig. 1A,B, it can be seen that the individual plant weight and yield of Guige 1 in different soil types rank as the following descending order: RYMS > BLS > SLS > YSCS > SLSW. There was no significant difference in the individual plant weight of Guige 1 among the five soil types, but there were differences in yield. Among them, the individual plant weight of RYMS is up to 3.20 kg and the theoretical yield reaches 3200.80 kg/667m^2^, showing significant differences from that in the other four soil types of theoretical yield. The yield in SLSW was significantly lower than that in the other three types of soil (Fig. 1A,B ). The type of soil restricts the growth and development of Guige 1, which in turn affects the individual plant weight and yield of *P. lobata*.

In Fig. 1C, it can be seen that the influence of five soil types on the contents of soluble sugar in Guige 1 ranks in the descending order: SLS > YSCS > SLSW > RYMS > BLS. There is no significant difference between SLS, YSCS and SLSW, but they are all significantly higher than RYMS and BLS. And, RYMS is also significantly higher than BLS. The order of the starch content of Guige 1 is BLS > RYMS > SLSW > SLS > YSCS, with the starch content in BLS being significantly higher than in the other four soil types. The starch content in RYMS is significantly higher than that in SLSW, SLS, and YSCS. Although there is no significant difference between the starch content in SLSW and SLS, both of them are significantly higher than that in YSCS. The descending order of the cellulose content of Guige 1 in five soil types ranks as YSCS > RYMS > SLS > SLSW > BLS. For the cellulose content, there is no significant difference between that in YSCS and RYMS, but the cellulose contents in YSCS and RYMS are significantly higher than that in SLS, SLSW and BLS. Although there is no significant difference between that in RYMS, SLS and SLSW, they are all significantly higher than that in BLS. There is no significant difference between the contents of the total flavonoids of Guige 1 in different soil types. The order of influence of five soil types on puerarin contents of Guige 1 from large to small is RYMS > BLS > SLSW > SLS > YSCS. There is no significant difference between the puerarin contents of Guige 1 in RYMS and BLS, but they are all significantly higher than that in the other three soil types, between which there is no significant difference in the puerarin contents.

Using fuzzy membership functions to comprehensively evaluate the quality of Guige 1 in five soil types of, the order of excellence is as follow: RYMS > BLS > SLS > YSCS > SLSW (Table 1).

**Table 1:** Weighted subordinate function values (WSFV) of five soil types on the yield and quality of Guige 1.

Soil types	Yield	Soluble sugar	Starch	Cellulose	Flavonoids	Puerarin	Average	Order
RYMS	1.00	0.49	0.39	0.81	0.33	1.00	0.67	1
SLSW	0.00	0.78	0.26	0.58	0.00	0.25	0.31	5
SLS	0.41	1.00	0.25	0.68	0.01	0.03	0.40	3
YSCS	0.32	0.87	0.00	1.00	0.11	0.00	0.38	4
BLS	0.52	0.00	1.00	0.00	1.00	0.94	0.58	2

### Analysis of total nitrogen, available phosphorus and available potassium in five soil types

**Figure 2:**
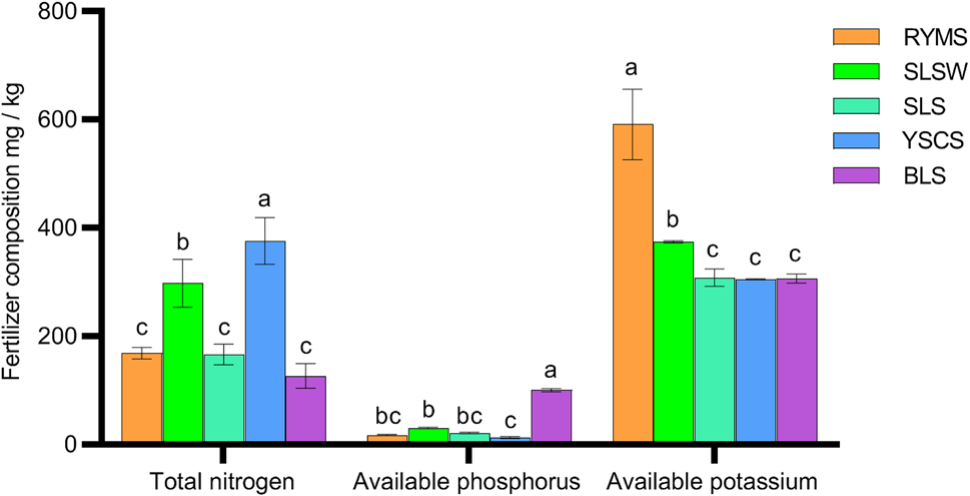
The content of total nitrogen, available phosphorus, and available potassium in five soil types. Different lowercase letters indicate significant difference (*p* < 0.05).

Figure 2 shows the content of total nitrogen, available phosphorus and available potassium in five soil types. It can be seen that the descending orders of total nitrogen, available phosphorus and available potassium contents in the five soil types are YSCS > SLSW > RYMS > SLS > BLS, BLS > SLSW > SLS > RYMS > YSCS, RYMS > SLSW > SLS > BLS > YSCS, respectively. The available potassium content in the RYMS reached 590.67 mg/kg, which was significantly different from the other four soil types. The individual plant weight and yield of Guige 1 were significantly higher than those of the other four soil types. When the concentration of K fertilizer is between 300 and 370 mg/kg, there is a very significant difference in the effective phosphorus content (100.09 mg/kg) of BLS compared to the other three soil types. The contents of starch, total flavonoids and puerarin of Guige 1 in BLS are significantly higher than those in the other three soil types. In SLS, SLSW and YSCS, the total nitrogen contents increase, while their individual plant weight and quality decreases.

The correlation analysis between the quality indicators and soil nutrients shows that except for the negative correlation between the starch accumulation in Guige 1and the content of nitrogen, available phosphorus and available potassium in the RYMS, the individual plant weight, cellulose, total flavonoids and puerarin content in Guige 1 are positively correlated with the content of total nitrogen, available phosphorus and available potassium in the RYMS and BLS. However, in the other soil types, the individual plant weight, cellulose, total flavonoids and puerarin content in Guige 1 are negatively correlated with the content of total nitrogen, available phosphorus and available potassium (Table 2). There is no significant correlation between the yield of Guige 1 and other factors in RYMS and SLS. In BLS, it is closely and positively correlated between the yield and the content of puerarin (0.936, *p* < 0.01), starch (0.976, *p* < 0.01) and cellulose (0.980, *p* < 0.01). This indicates that the nutrient changes in different soil types are related to the yield and quality formation of Guige 1. The content of high available phosphorus and available potassium in RYMS and BLS may affect the quality formation of Guige 1, while waterlogged or hardened land is not conducive to the utilization of nitrogen fertilizer. Overapplication of nitrogen fertilizer is not conducive to the growth, development and quality formation of Guige 1.

**Table 2:** Relationships between Yield of Guige 1 and Different Soil Types.

Soil type	Single plant weight	Soluble sugar	Starch	Cellulose	Total flavonoids	Puerarin	Total nitrogen	Available phosphorus	Available potassium
RYMS	1.000**	−0.299	−0.197	0.425	.a	0.317	0.114	0.502	0.482
SLSW	1.000**	0.708	−0.496	−0.732*	0.291	−0.740*	−0.739*	−0.325	−0.527
SLS	1.000**	−0.771	0.493	0.602	−0.442	−0.769	−0.495	−0.628	−0.714
YSCS	1.000**	−0.243	−0.227	0.775	−0.766	−0.692	−0.706	−0.815*	−0.692
BLS	1.000**	0.375	0.976**	0.980**	0.369	0.938**	0.685	0.442	0.075

* *p* < 0.05; ** *p* < 0.01; a: Because at least one variable is a constant, it cannot be calculated.

### Analysis of endophytes in Guige 1 from five soil types

**Figure 3:**
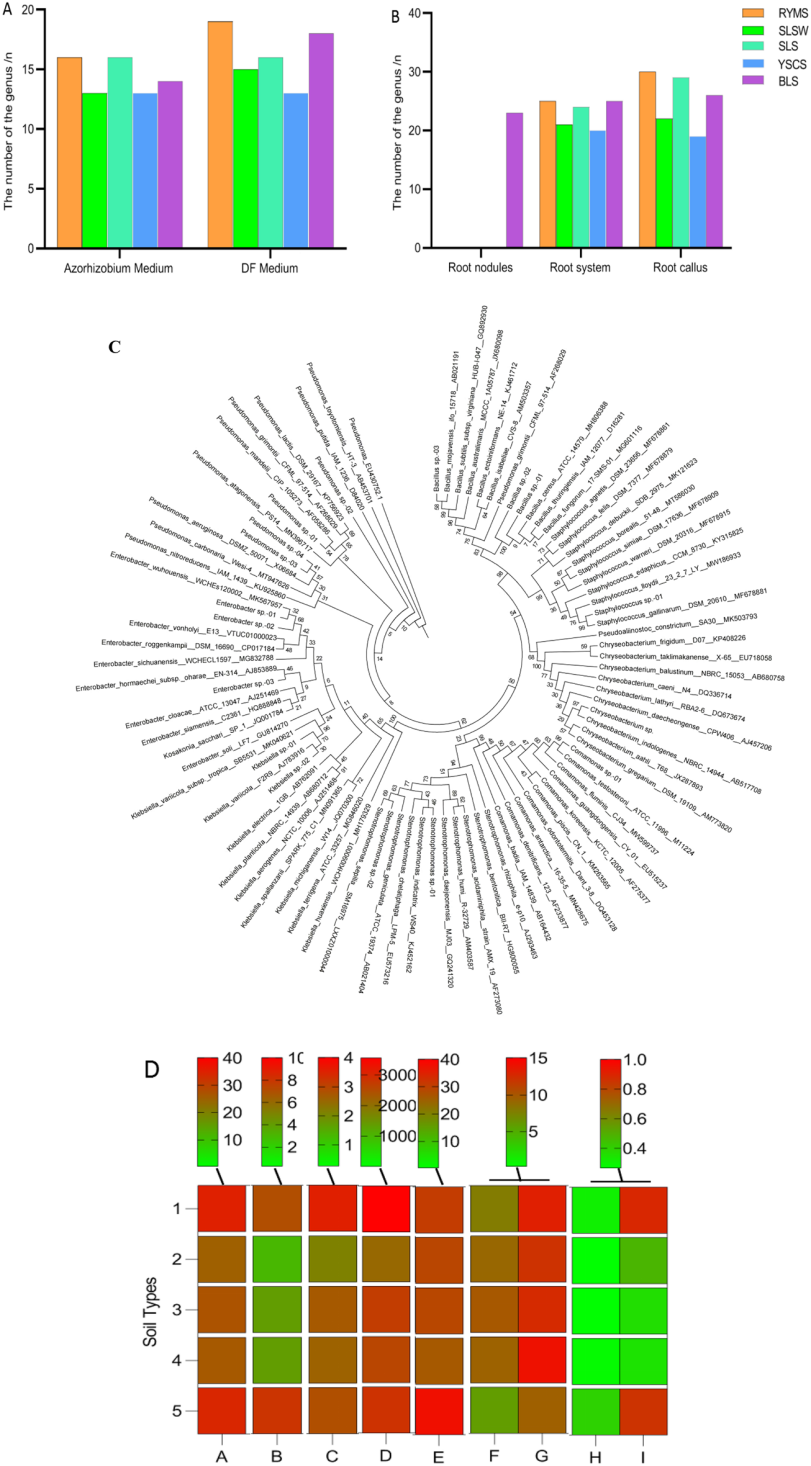
Comparison of the types and quantities of endophytic fungi in *Pueraria lobata* of different soil types. Number of endophytic bacterial strains isolated from different culture medium (**A**) and parts (**B**). Perform phylogenetic tree analysis on the top 5 strains with the highest homology to NCBI blast (**C**). Heat map of the number of endophytic and dominant bacterial species in Guige 1 of five soil types and their relationship with the yield and quality of Guige 1. Among them, 1–5 represents RYMS, SLSW, SLS, YSCS and BLS, respectively. A-I represents the number of endophytic species, the number of dominant endophytic species, individual plant weight, yield, starch content, soluble sugar content, cellulose content, total flavonoid content, and puerarin content, respectively. The redder the color, the higher the correlation (**D**).

Using DF and nitrogen-fixing rhizobial solid culture medium as isolation medium, we isolated and identified endophytes in the root tissues including root nodules, root systems and root calli of Guige 1 cultivated in five soil types. Based on the results of 16 S rRNA molecular identification, a total of 35 endophytes were isolated, of which 16 were isolated from nitrogen fixation medium, with 7 dominant strains, including *Stenotrophomonas*, *Klebsiella*, *Bacillus*, *Pseudomonas*, *Chryseobacterium*, *Enterobacter* and *Staphylococcus*; 19 species were isolated from DF culture medium, with 8 dominant strains, including *Stenotrophomonas*, *Klebsiella*, *Bacillus*, *Pseudomonas*, *Chryseobacterium*, *Enterobacter*, *Staphylococcus* and *Comamonas* (Fig. 3A,C , and Table 3). There is an additional species of *Comamonas* compared to nitrogen fixation medium.

**Table 3:** Registration Form of Dominant Strains Isolated from Different Parts (Root Nodules, Roots, and Callus) of Guige 1 in Five Soil Types on Two Different Media (Nitrogen Fixation and DF Media).

Soil type	Nitrogen fixation medium				DF medium			
	Separation site	Strain number	Percentage / %	Dominant bacteria genus	Separation site	Strain number	Percentage / %	Dominant bacteria genus
BYMS	Root nodules	/	/	/	Root nodules	/	/	/
	Root system	N7	58.21	*Enterobacter sp.-01*	Root system	DF5	48.48	*Klebsiella sp.-01*
		N8	26.87	*Pseudomonas sp.-01*		DF6	34.85	*Staphylococcus sp.-01*
	Root callus	N26	33.67	*Klebsiella sp.-01*	Root callus	DF22	33.73	*Bacillus sp.-01*
		N27	30.61	*Bacillus sp.-01*		DF23	27.71	*Stenotrophomonas sp.-01*
		N28	26.53	*Chryseobacterium sp.-01*		DF24	26.51	*Chryseobacterium sp.-01*
SLSW	Root nodules	/	/	*/*	Root nodules	/	/	*/*
	Root system	N13	64.52	*Enterobacter sp.-02*	Root system	DF9	69.43	*Enterobacter sp.-02*
	Root callus	N32	59.82	*Bacillus sp.-02*	Root callus	DF28	70.59	*Pseudomonas sp.-03*
SLS	Root nodules	/	/	*/*	Root nodules	/	/	*/*
	Root system	N16	54.02	*Stenotrophomonas sp.-01*	Root system	DF12	71.77	*Pseudomonas sp.-04*
	Root callus	N36	70.71	*Bacillus sp.-01*	Root callus	DF34	86.90	*Klebsiella sp.-02*
YSCS	Root nodules	/	/	*/*	Root nodules	/	/	*/*
	Root system	N18	48.61	*Klebsiella sp.-01*	Root system	DF15	68.52	*Enterobacter sp.-02*
	Root callus	N41	46.59	*Bacillus sp.-02*	Root callus	DF37	52.08	*Bacillus sp.-02*
BLS	Root nodules	N1	40.74	*Enterobacter sp.-03*	Root nodules	DF2	67.05	*Pseudomonas sp.-02*
		N3	31.48	*Staphylococcus sp.-01*		/	/	*/*
	Root system	N20	43.59	*Bacillus sp.-03*	Root system	DF19	43.75	*Bacillus sp.-03*
		N21	34.62	*Chryseobacterium sp.-01*		DF20	31.25	*Staphylococcus sp.-01*
	Root callus	N46	30.17	*Klebsiella sp.-02*	Root callus	DF43	30.34	*Pseudomonas sp.-02*
		N47	25.86	*Chryseobacterium sp.-01*		DF44	24.72	*Stenotrophomonas sp.-02*
		N48	21.55	*Pseudomonas sp.-02*		DF45	22.47	*Chryseobacterium sp.-01*
		N49	21.55	*Enterobacter sp.-03*		DF46	20.22	*Comamonas sp.-01*

From the data (Fig. 3A,C, and Table 3) analysis, it can be seen that a total of 30 bacterial genera and 7 dominant bacterial genera were found in Guige 1 cultivated in RYMS, including *Enterobacter*, *Pseudomonas*, *Klebsiella*, *Bacillus*, *Staphylococcus*, *Chryseobacterium* and *Stenotrophomonas*. A total of 22 bacterial genera and 3 dominant bacterial genera were found in Guige 1 cultivated in SLSW, namely Enterobacter, Pseudomonas, and Bacillus. A total of 28 bacterial genera and 4 dominant bacterial genera were found in Guige 1 cultivated in SLS, including *Pseudomonas*, *Klebsiella*, *Bacillus* and *Stenotrophomonas*. A total of 24 genera of bacteria were found in Guige 1 cultivated in YSCS, with 3 dominant genera, namely *Enterobacter*, *Klebsiella*, and *Bacillus*. A total of 31 bacterial genera and 8 dominant bacterial genera have been found in Guige 1 cultivated in BLS, among which 7 species are consistent with that of RYMS and 1 species is unique to that of BLS, namely Comamonas.

Comparing the endophytic bacteria in the three different parts of Guige 1 (Fig. 3B,C, and Table 3), root nodules were only found in the BLS, and 23 bacterial species were isolated from that. Among the 23 bacterial species above, there were 3 dominant strains, namely *Enterobacter*, *Pseudomonas* and *Staphylococcus*. 25 bacterial species were isolated from the root system, with 7 dominant strains including *Enterobacter*, *Pseudomonas*, *Klebsiella*, *Bacillus*, *Staphylococcus*, *Chryseobacterium* and *Stenotrophomonas*. 30 bacterial species were isolated from callus tissues, with 7 dominant strains including *Enterobacter*, *Pseudomonas*, *Klebsiella*, *Bacillus*, *Chryseobacterium*, *Stenotrophomonas* and *Comamonas*. The species and quantity showed tissue differences, namely, callus tissue > root system > root nodule.

From the analysis of heat map (Fig. 3D), it can be seen that there are abundant endophytes and dominant bacterial species isolated from Guige 1 of BLS and RYMS, while there are relatively few endophytic and dominant bacterial species isolated from Guige 1 of SLS, YSCS and SLSW. The more endophytic populations and dominant bacteria from Guige 1 of RYMS and BLS were isolated, the higher individual plant weight, yield and content of starch and puerarin in Guige 1 are correspondingly measured. The numbers of endophytic populations and dominant genera in Guige 1 of RYMS and BLS are positively correlated with the individual plant weight, yield and content of starch and puerarin in Guige 1.

### Effects of different fertilizers on the yield and quality of Pueraria lobata

**Figure 4:**
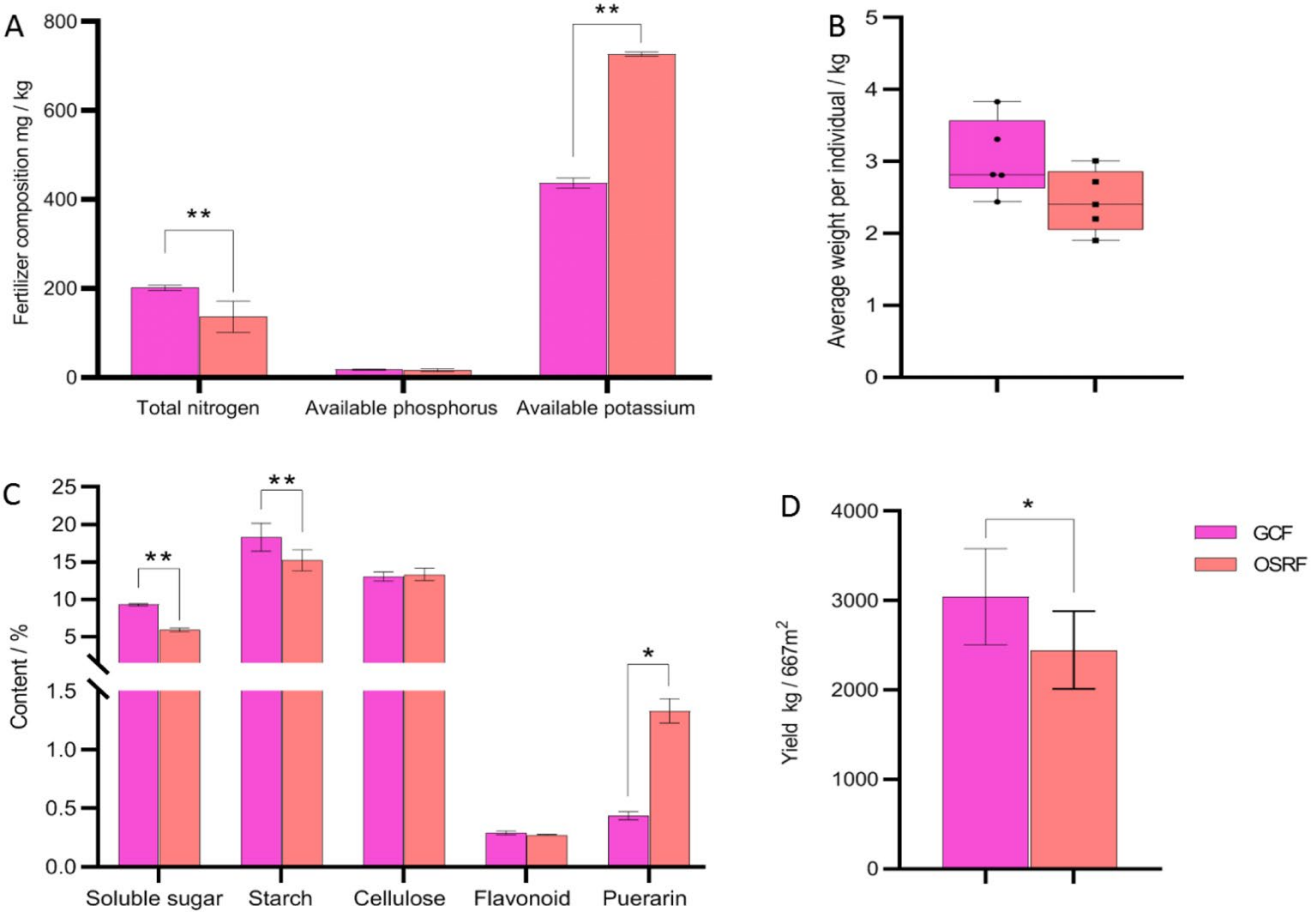
The effect of two fertilizers on the yield and quality of Guige 1. (**A**) The content of total nitrogen, available phosphorus and available potassium with two fertilizer types treated; (**B**, **D**) The average plant weight (**B**) and yield (**D**) of Guige 1 with two fertilizers treated; (**C**) The effects of two fertilizers on the quality of soluble sugar, starch, cellulose, total flavonoids and puerarin in Guige 1. *p < 0.05; **p < 0.01.

In order to investigate the effects of GCF and OSRF on *Pueraria lobata* production, we conducted a comparative experiment on the yield and quality of Guige 1 with two different fertilizers in RYMS. Measurements of total nitrogen, available phosphorus and available potassium content were conducted on the soil, and it was found that the total nitrogen content of the soil treated with GCF was significantly higher than that with OSRF, contrary to the content of available potassium, while there was no significant difference in the content of available phosphorus (Fig. 4A). The total nitrogen content in the GCF treatment reached 201.36 mg/kg, which was significantly higher than that in the OSRF treatment (136.16 mg/kg), with a difference of 65 mg/kg. The content of available potassium reached 436.89 mg/kg, significantly lower than that of OSRF treatment (726.78 mg/kg), with a difference of 290 mg/kg.

The experimental results showed that the single plant weight and yield of Guige 1 treated with GCF reached 3.04 kg and 3042 kg/667m^2^, which was significantly higher than that in the OSRF treatment with average single plant weight of 2.45 kg and the yield of 2446 kg/667m^2^ (Fig. 4B,D). Further quality comparison on Guige 1 found that the soluble sugar and starch content of Guige 1 treated with GCF were significantly higher than that with OSRF, contrary to the puerarin content, while there was no significant difference in the content of cellulose and total flavonoids (Fig. 4C). Among them, the glucose and starch contents of Guige 1 treated with GCF reached 9.40% and 18.29%, respectively, which were significantly higher than those of OSRF treatment (6.10% and 15.19%). However, the content of puerarin (0.44%) was significantly lower than that (1.33%) treated with OSRF. The results suggest that GCF can significantly promote the growth and development of Guige 1 in RYMS, increase the weight of each plant, yield and the content of soluble sugar and starch of *P. lobata*. Thus, OSRF is more suitable for the accumulation of puerarin in Guige 1.

## Discussion

There are differences in nutrients and physicochemical properties among different soil types. Reasonable fertilization based on specific soil conditions can improve soil quality and promote the growth and development of plants. Previous studies have shown that the comprehensive fertility of different soil types, is in the descending order of purple soil > lime soil > yellow-brown soil > rice paddy soil > moisture soil. Moisture soil has the lowest fertility and the worst fertilizer retention ability, but the effective phosphorus content is relatively high. Thus, organic fertilizer and nitrogen potassium fertilizer can be increased to control the application of phosphorus fertilizer. The soil fertility and fertilizer retention capacity of rice paddy soil take second place, but the content of available phosphorus and total nitrogen is relatively rich. Hence, increasing the application of phosphorus fertilizer and controlling the use of potassium fertilizer to improve soil structure, and then improve the quality of plants^22^. The quality of plants is closely related to the nitrogen, phosphorus and potassium in the soil. The starch content in the roots of *P. lobata* is significantly and positively correlated with the available potassium and phosphorus content in the soil^23^. Adequate potassium content can accelerate the synthesis of monosaccharides into sucrose and starch, and then improve the yield and quality of starch crops^24^. Nitrogen is the central and active nutrient required by plants, and it is an important environmental factor for plant photosynthesis, growth and development^25,26^. However, high nitrogen content in the soil can easily cause overgrowth of the aboveground parts of plants, which can lead to a decrease in economic benefits and even yield for rhizome based medicinal materials^27^. At the same time, high nitrogen can actually weaken or even inhibit plant photosynthesis^28^. The results of this study confirmed previous research findings. In both RYMS and BLS, there are much available potassium and available phosphorus and relatively high individual plant weight, yield and the content of starch, total flavonoids and puerarin in Guige 1. However, soil compaction or flooding can lead to the accumulation of total nitrogen, promote overgrowth of aboveground parts, inhibit the development and expansion of underground roots, and thus lead to a decrease in individual plant weight and yield of Guige 1. As a leguminous plant, *P. lobata* itself has the synergistic effect of producing nodules and rhizobia to fix nitrogen in the atmosphere. Therefore, when planting *P. lobata* in RYMS or BLS, in addition to applying a small amount of nitrogen fertilizer as the base fertilizer, it is advisable to not or apply less nitrogen fertilizer in later management.

Research has shown that endophytes mainly promote plant growth and development through two pathways. Firstly, endophytes directly promote plant growth and development through biological nitrogen fixation, production of iron carriers, phosphorus dissolution, synthesis of specific enzymes or secretion of substances such as auxin, cytokinin, gibberellin and so on. The second pathway is to indirectly promote plant growth and development by promoting the synthesis of various plant growth hormones by the host plant itself, improving the utilization rate of nutrients such as nitrogen, phosphorus and potassium, inducing the host plant to synthesize some small molecule substances or enzymes to increase the sensitivity of the plant to biotic and abiotic stress^29^. The previous study showed that *Bacillus* is an absolute dominant bacterium in the roots^30^, occupying an important position in promoting endophytic bacteria and possessing the ability to secrete biomass to improve plant growth^31,32^. In addition, *Bacillus*, *Pseudomonas*, and *Agrobacterium* are widely recognized as phosphorus solubilizing microorganisms in promoting endophytes growth. A literature also found that the dominant bacterial groups isolated from Guige 8 included *Bacillus*, *Pseudomonas*, *Enterobacter*, and *Agrobacterium*, all of which have characteristics of nitrogenase activity, secretion of IAA, ferritin and calcium phosphate^33^. Among them, *Pseudomonas*, *Agrobacterium* and *Klebsiella* have also been detected to possess the nifH gene.We conducted endophytic isolation and identification experiments on Guige 1 from five types of soil. And we found that the types and quantities of endophytic bacteria in RYMS and BLS were similar, significantly higher than the other three soil types, including dominant bacterial groups of *Bacillus*, *Enterobacter*, *Pseudomonas*, *Klebsiella*, *Staphylococcus* and *Chryseobacterium*. The dominant bacterial groups such as *Staphylococcus* and *Chryseobacterium* have been demonstrated by the member of our research group^33^, to have 2-4 types of growth promoting effects. This may be one of the reasons for the high yield and good quality of Guige 1 in RYMS and BLS.

GCF contain two or more elements required by crops, with high nutrient content, and can provide a balanced supply of nutrients required by crops^34^. Fertilization has a significant yield increase effect, but the fertilizer effect is time limited, requiring multiple fertilization. OSRF are rich in multiple types of bacteria, which can effectively enrich the types of microorganisms and endophytic bacteria in the soil, maintain good soil physicochemical properties, slowly release effective nutrients and gradually be absorbed and utilized by crops^35,36^. The fertilizer efficiency can be maintained for several months. Ding Xiaoxia et al.^37^ applied OSRF to meet the nutrient demand of corn at all reproductive stages, increased the number of ear grains and increased the grain weight, so as to achieve the purpose of saving cost and increasing production. Based on the RYMS, this study found that GCF is more suitable for the production of Guige 1, which can significantly increase its yield. OSRF can effectively improve the quality of Guige 1, enhance its medicinal value. The main component of starch in *P. lobata* tubers^38^gradually increases throughout the entire growth period, with a rapid accumulation stage from June to October, and then slightly increased or became stable after October^39^. Nitrogen is an important part of nucleic acids, proteins, chlorophyll and other substances in plants. Reasonable application of nitrogen fertilizer can significantly improve the rate of plant photosynthesis and promote plant growth^40^. The accumulation of starch during the root tuber development stage requires nitrogen, phosphorus and potassium, which can be well met by GCF. Therefore, the weight and yield of *P. lobata* are relatively high. The slow release of nutrients from OSRF results in a relatively slow growth and development of *P. lobata*. At the same time, the soil is rich in microbial species, which is conducive to the accumulation of substances such as puerarin and isoflavones, and thereby improves the quality of medicinal materials.

## Methods

### Plant and soil materials

The experiment used the variety of Guige 1 (*Pueraria Montana var. thomsonii*) selected by our research group. Guige 1 was planted in early April of the same year and three plants were randomly selected in early July. The root nodules, roots and cut calli of cutting branches were taken as experimental materials. At the end of December, 3 plants were randomly collected for measurement of individual plant weight and yield, and quality analysis was conducted on the root tubers. Repeat each experiment three times.

Five types of soil were collected from five villages in Guangxi, including RYMS collected from Bantang Village, Lingyun County; SLSW collected from Yangcun, Lingyun County; SLS collected from Naxi Tun, Lingyun County; YSCS collected from Naya Tun, Lingyun County; and BLS collected from Shixia Village, Teng County. Each type of soil is randomly collected from 3 locations at a certain distance, based on the terrain, and each location is collected using the five-points sampling method. Five sub samples of surface soil are collected and mixed as one sample^41^. At the same time, we record the soil type, planting density and other information of the samples, and refer to the "Classification and Codes for Chinese Soil" of the People's Republic of China GB/T17296-2009^42^.

### Fertilizer experiment

This experiment was conducted on the RYMS from Bantang Village, Lingyun County. The GCF treatment uses ordinary domestic compound fertilizer (Zhongnong High Concentration Potassium Sulfate Compound Fertilizer) with a N: P: K ratio of 17:17:17 and a dosage of 160 kg/667m^2^. OSRF treatment formula: neutral general compound fertilizer, pH ≥ 6, N: P: K ratio 17:17:17, dosage of 30 kg/667m^2^; neutral medium element fertilizer, with a dosage of 10 kg/667m^2^; trace element fertilizer, with a dosage of 5 kg/667m^2^; alkaline calcium magnesium phosphorus (pH ≥ 8.5), with a dosage of 50 kg/667m^2^; 500 million/g GDC (salt reducing and fertilizer reducing) strain, with a dosage of 20 kg/667m^2^; 1 billion/g of dry grass (potassium phosphate), with an amount of 40 kg/667m^2^. Two types of fertilizers were applied as the base fertilizer in April at the same year, respectively. With GCF applied twice at the middle and later stage of the experiment, OSRF treatment was not applied anymore. The field management of the two groups is the same.

### Soil analyses methods

The soil sample is dried in the shade under natural conditions, removing stones and plant debris, passing through a 2 mm nylon sieve and using the quartering method to select 1 kg sample for backup. The determination of soil total nitrogen content refers to the determination of forest soil total nitrogen by the National Forestry Administration LYIT 12-1999^43^. The determination of soil available phosphorus is carried out using efficient testing techniques based on conventional soil available phosphorus (OIsen method) analysis by Yang Liping et al.^44^. The determination of soil available potassium is carried out using the Ministry of Agriculture of the People's Republic of China NY/T1849-2010 and NY/T1848-2010^45,46^.

### Quality measurement of Guige 1

After washing and drying the collected Guige 1 samples, we cut the middle part of the roots into thin slices, blanch them at 105 ℃ for 30 minutes, dry them at 55 ℃ to constant weight, then crush and pass them through a 100 mesh sieve for backup. The soluble total sugar and starch content were determined using the anthrone colorimetric method^47^. The crude fiber content was determined according to the method of a previous study^48^. The total flavonoid content was determined according to the methods of a previous research^49^. The content of puerarin was determined by high-performance liquid chromatography^50,51^, with the chromatograph being Waters e2695 and the chromatographic column being Symmetry C18 (4.6mm × 250.0 mm, 5 μ m). The mobile phase is methanol (chromatographic grade) -0.1% acetic acid water (30:70). The flow rate is 1.0 mL/min. The detection wavelength is 250 nm. Column temperature is 30 ℃.

### Isolation, purification, and identification of endophytic bacteria

Isolation and purification of endophytic bacteria were conducted referring to a previous literature^52^, by disinfecting the surface of the sample and grounding the material with phosphate buffer under sterile conditions to form a homogenate. The material was inoculated onto DF solid culture medium^53^ and nitrogen-fixing rhizobia solid culture medium (Qingdao High tech Industrial Park Haibo Biotechnology Co., Ltd.), respectively. Place the culture dishes in a 28 ℃ and light incubator for7 days, observe and record the size and appearance of the bacteria daily. Simultaneously select representative single colony bacterial species for preservation and species identification. The bacterial strain is punctured and stored at room temperature in solid culture medium or inoculated on LB liquid culture medium, and then stored in a refrigerator at -80 ℃ with 30% glycerol added.

Identification of endophytic bacteria species. Advanced extraction of bacterial genomic DNA (Kangwei Century Biotechnology Co., Ltd.) was conducted, followed by PCR amplification using the universal primers F8 (5 ′ - AGAGTTTGATCCTGGCTCAG-3 ′) and R1541 (5-AAGGAGGTGATCCAGCCGCA-3 ′) for prokaryotic 16S rRNA genes. PCR reaction system (50 μL): template DNA, 2 μL; F8 and R1541 primers (10 μmol/L, 1μL, respectively); ddH2O, 21 μL; 2 × Taq PCR Master Mix, 25 μL. PCR reaction conditions: 95 ℃ for 5 minutes; 94 ℃ for 30 seconds; 55 ℃ for 30 seconds; 72 ℃ for 1.5 minutes, cycling 30 times; 72 ℃ 10 Min, 16 ℃ 10 Min. 5 μL PCR reaction products were loaded in 1% agarose gel, 120 V electrophoresis for 20 minutes. And then the PCR product fragments of each gene with correct size, bright and clear bands were sent to Shenggong Biotechnology (Shanghai) Co., Ltd. for sequencing. Submit the 16S rRNA gene sequence of the tested strain to the GenBank database for homologous BLAST alignment analysis and construct a phylogenetic evolution tree using MEGA 7 software.

### Statistical analyses

Use Excel 2007, SPSS 11.5 and GraphPad Prism 9.3 software to analyze and process the data, using Duncan's new complex range method for significance analysis. Using the membership function method of fuzzy mathematics to comprehensively analyze the quality differences of Guige 1. Calculation method of membership function:

The formula for calculating the membership function value:

1 $$ {\text{R }}\left( {{\text{Xi}}} \right) \, = \, \left( {{\text{Xi}} - {\text{ Xmin}}} \right)/\left( {{\text{Xmax }} - {\text{ Xmin}}} \right) $$

Formula for calculating the value of the inverse membership function:

2 $$ {\text{R }}\left( {{\text{Xi}}} \right) \, = { 1} - \, \left( {{\text{Xi }} - {\text{ Xmin}}} \right)/\left( {{\text{Xmax }} - {\text{ Xmin}}} \right) $$

In the formula, Xi is the measured value of the indicator, and Xmin and Xmax are the minimum and maximum values of a certain indicator for all test materials.

### Plant guideline statement

The appropriate permissions and/or licences for collection of plant or seed specimens. The variety of Guige 1 (*Pueraria Montana var. Thomsonii*) used in this experiment was selected and cultivated by the project host Professor Wang Aiqin in 2014, and we received permission, support and guidance from Professor Wang Aiqin throughout the entire experiment process.

Meantime, experimental research and field studies on plants (either cultivated or wild), including the collection of plant material,we comply with the IUCN Policy Statement on Research Involving Species at Risk of Extinction and the Convention on the Trade in Endangered Species of Wild Fauna and Flora.

### Supplementary Information

Supplementary Information.

## Data Availability

The datasets used and/or analysed during the current study available from the corresponding author on reasonable request.
